# Case Report: A first case of desmin-related myofibrillar myopathy due to inheritance from a confirmed mosaic asymptomatic carrier

**DOI:** 10.3389/fgene.2025.1597851

**Published:** 2025-06-18

**Authors:** Jelle Vlaeminck, Sophie Uyttebroeck, Elke De Schutter, Ann Cordenier, Shauni Wellekens, Erwin Ströker, Kelly De Rooms, Christine Helsen, Frederik J. Hes, Philippe Giron

**Affiliations:** ^1^ Centre for Medical Genetics, Research Group Genetics, Reproduction and Development (GRAD), Clinical Sciences, Universitair Ziekenhuis Brussel (UZ Brussel) - Vrije Universiteit Brussel (VUB), Brussels, Belgium; ^2^ Department of Neurology, Center for Neurosciences, Universitair Ziekenhuis Brussel (UZ Brussel) - Vrije Universiteit Brussel (VUB), Jette, Belgium; ^3^ Department of Respiratory Medicine, Universitair Ziekenhuis Brussel (UZ Brussel) - Vrije Universiteit Brussel (VUB), Jette, Belgium; ^4^ Heart Rhythm Management Centre, Universitair Ziekenhuis Brussel (UZ Brussel) - Vrije Universiteit Brussel (VUB), Jette, Belgium

**Keywords:** desmin-related myopathy, mosaicism, *DES*, c.1216C>T, R406W, Arg406Trp, case report

## Abstract

Desmin-related myofibrillar myopathy is a hereditary disorder caused by pathogenic variants in the *DES* gene (MIM*125660), altering desmin, a muscle-specific intermediate filament which is crucial for sarcomere integrity. This condition presents with skeletal myopathy, cardiomyopathy, and conduction abnormalities. Genetic counselling for index patients and their family members is complicated by variable expressivity, incomplete penetrance, and *de novo* occurrence. Mosaicism in asymptomatic parents can obscure inheritance patterns, particularly when low-grade mosaic variants in blood may be missed. In case of *DES*, mosaic carriership has not been described before. We describe a case of a 24-year-old female diagnosed with desmin-related myopathy due to a heterozygous pathogenic NM_001927.4 (*DES*):c.1216C>T, p.Arg406Trp variant. Cascade testing using targeted Sanger sequencing of her asymptomatic parents suggested the mother is a mosaic carrier of the pathogenic variant, which was confirmed though next-generation sequencing. The proband’s siblings did not carry the *DES* c.1216C>T variant. We report the first documented case of mosaic carriership of a pathogenic *DES* variant in an asymptomatic individual and subsequent inheritance by the offspring, leading to desmin-related myopathy. This report highlights the importance of cascade testing in hereditary disorders with a focus on mosaicism, even when the index’s biological parents are asymptomatic, and *de novo* emergence is suspected.

## 1 Introduction

Desmin-related myofibrillar myopathy is a progressive, hereditary disorder, that primarily affects skeletal, cardiac, and respiratory muscles. The condition is caused by pathogenic aberrations in the desmin gene (*DES,* MIM *125660), which encodes a muscle-specific type III intermediate filament protein. Desmin plays a crucial role in regulating sarcomere architecture, ensuring proper muscle function and structural integrity (Brodehl et al., 2018). Pathogenic variants in *DES,* which can be inherited or occur *de novo*, disrupt desmin filament formation, leading to defects in muscle cells, particularly in intercalated discs, which are vital for muscle contraction and communication. This is not due to reduced desmin production, as expression is often not significantly decreased, but rather a dominant negative effect, where the mutated desmin interferes with the normal function of wild type desmin. This disruption affects essential cellular processes, including protein interactions, mitochondrial function, and the filament network (Clemen et al., 2013).

The disease often manifests with progressive muscle weakness and atrophy, initially affecting the proximal skeletal muscles (e.g., shoulders and hips) and later involving distal muscles (e.g., hands and feet). Involvement of bulbar muscles can lead to dysphagia (difficulty swallowing) and dysarthria (difficulty speaking), while respiratory muscles can be impaired, leading to restrictive lung disease and respiratory insufficiency (Goldfarb et al., 2004). Cardiac involvement is common and typically presents with dilated cardiomyopathy or less frequently arrhythmogenic right ventricular cardiomyopathy, leading to heart failure and arrhythmias. These arrhythmias include ventricular arrhythmias and atrial fibrillation, significantly increasing the risk of sudden cardiac death, often at a young age (Brodehl et al.,2018; Wilde et al., 2022).

Desmin-related myopathy follows an autosomal dominant inheritance pattern, although cases with autosomal recessive inheritance patterns have been described (Onore et al., 2022). Furthermore, the disease exhibits incomplete penetrance and variable expressivity, meaning that not all individuals with the mutation will develop symptoms, and that symptom severity varies. The variability in expression is partially explained by the location of the genetic variants within the *DES* gene (Bär et al., 2007; Goldfarb et al., 2008; Clemen et al., 2013).

In this report, we describe a case of a young woman diagnosed with desmin-related myopathy after genetic testing. Targeted sequencing of the pathogenic *DES* c.1216C>T variant in peripheral leukocytes of the asymptomatic parents was inconclusive. Next-generation sequencing (NGS) showed mosaic carriership in the mother of the same DES variant, leading to further family cascade testing.

## 2 Case description

**FIGURE 1:**
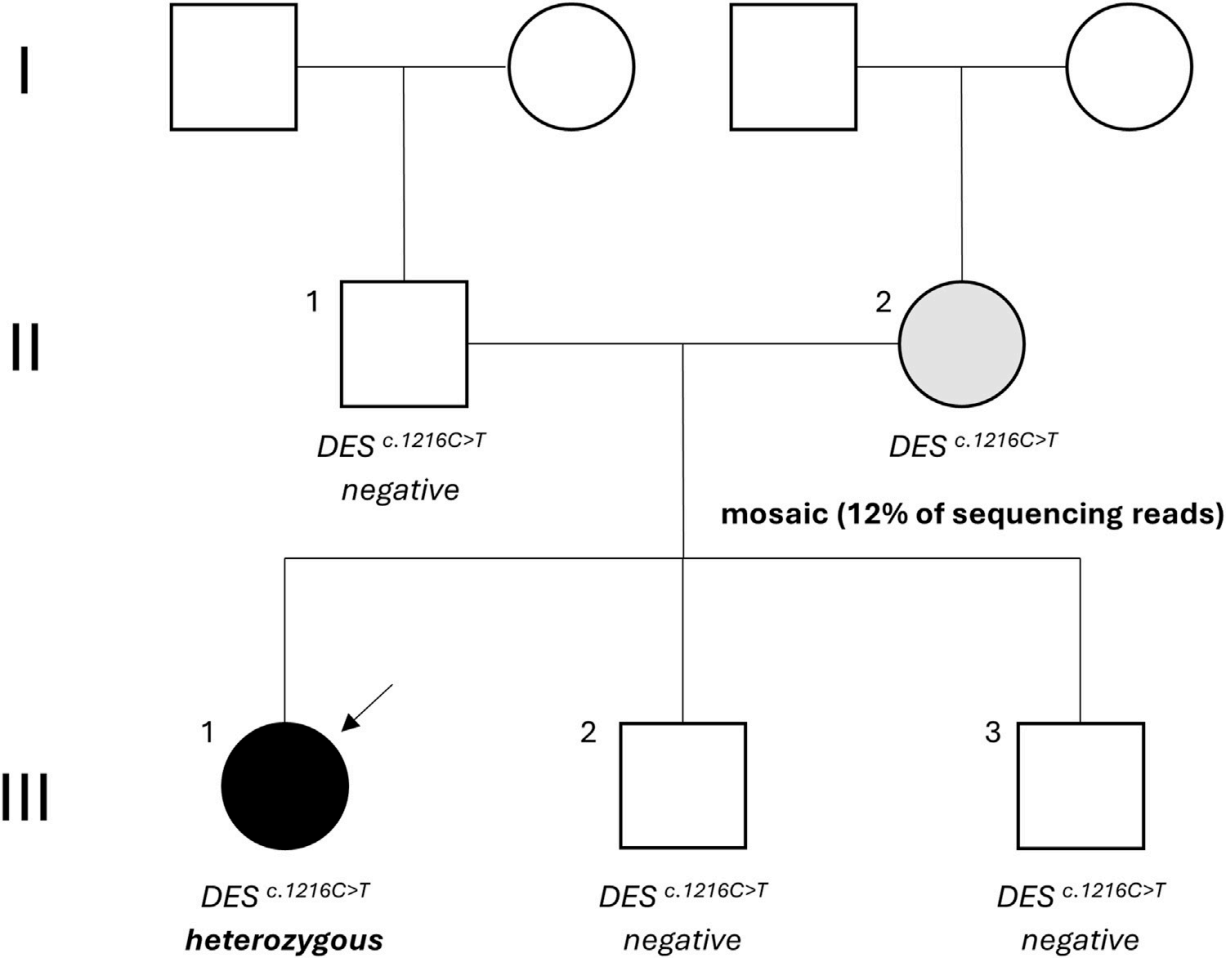
Family tree. The proband is indicated by the arrow and status of the NM_001927.4 (*DES*):c.1216C>T variant is mentioned. Black: desmin-related myofibrillar myopathy, grey: asymptomatic carrier, white: unaffected. *DES*: desmin.

The proband was a 24-year-old female (Figure 1, III-1) with a history of atrioventricular conduction disorder and fasciculoventricular bypass tracts. Prior to this diagnosis in 2016, the proband had no cardiovascular symptoms and maintained a healthy, sportive lifestyle. At the age of 16, she suffered an unprovoked syncope with visual disturbances, heat flashes, dizziness, and loss of consciousness. Upon cardiac evaluation, electrocardiogram showed ventricular pre-excitation, suggesting hypertrophic cardiomyopathy. Transthoracic ultrasound revealed a borderline proximal septal thickness of 9 mm. A bicycle ergometry test was planned but had to be aborted due to vasovagal syncope with bradycardia. During a provocative ajmaline test, the proband suffered a cardiopulmonary arrest, but was resuscitated successfully. A double-chamber pacemaker (DDD pacemaker, Medtronic, United States) was implanted after which the proband had no further cardiac symptoms and attended regular follow-up. Familial anamnesis showed no familial cardiovascular illnesses apart from the maternal grandmother using a beta-blocker for tachycardia (Figure 1). Given the young age of the proband, genetic testing for hereditary cardiovascular diseases was proposed and the proband was referred to a clinical geneticist.

## 3 Diagnostic assessment

### 3.1 Genetic workup

Following informed consent, leukocyte DNA was extracted from whole peripheral blood and whole exome sequencing (WES) performed on a Novaseq 6000 (Illumina, Inc., United States) platform. An *in silico* hereditary cardiac disorder gene panel, consisting of 279 genes, was analysed using GRCh37. Three missense variants, one in *DES* and two in *TTN*, were identified (Table 1).

**TABLE 1:** Variant features of detected variants in the proband.

cDNA	NM_001927.4 (*DES*): c.1216C>T	NM_001267550.2 (*TTN*): c.44750A>G	NM_001267550.2 (*TTN*): c.39835A>G
dbSNP	rs121913003	NA	rs2062656094
gDNA	g.220286254C>T	g.179489257T>C	g.179514604T>C
Location	Exon 6	Exon 242	Exon 211
pNomen	p.Arg406Trp	p. (Glu14917Gly)	p. (Lys13279Glu)
Coding Effect	Missense	Missense	Missense
Variant Allele Frequency	49.1%	42.5%	42.1%
Median Allele Frequency (gnomAD v4.1.0)	NA	0.0004340%	0.0006827%
Clinvar ID	16826	NA	892694
Clinvar classifications	Pathogenic (10), Likely pathogenic (1)	NA	Uncertain significance (5)
REVEL score^*^	0.807	0.243	0.166
AlphaMissense score^§^	0.993	0.152	0.124
ACMG Criteria	PS2-PS3^#^-PM2-PM5	PM2	PM2
ACMG pathogenicity class	Pathogenic	Variant of uncertain significance	Variant of uncertain significance

NA: not available, ^*^: Ioannidis et al., 2016, ^§^: Cheng et al., 2023, ^#^: Herrmann et al., 2020.

**FIGURE 2:**
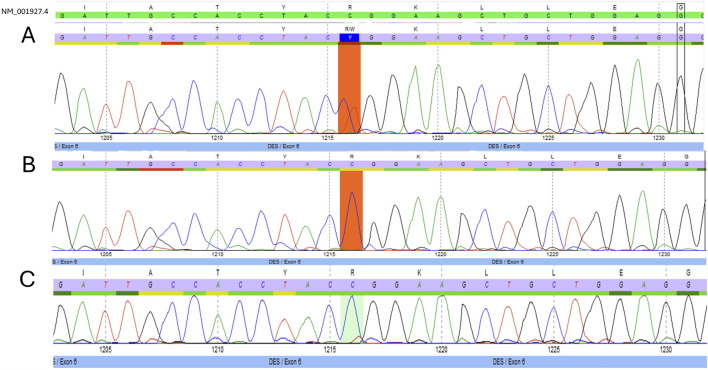
Electropherograms. Targeted Sanger sequencing of exon six of the desmin gene (*DES)* with position c.1216 marked. **(A)** Presence of the NM_001927.4 (*DES*):c.1216C>T variant in heterozygous form in the proband. **(B)** Absence of the NM_001927.4 (*DES*):c.1216C>T variant in the proband’s father. **(C)** Suspected mosaicism of the NM_001927.4 (*DES*):c.1216C>T variant in the proband’s asymptomatic mother.

The NM_001927.4 (*DES*):c.1216C>T, p.Arg406Trp variant was described several times with *de novo* occurrence in patients with severe, early onset cardiomyopathy with or without additional myopathy (Dalakas et al., 2000; Park et al., 2000; Dagvadorj et al., 2004). Additionally, a functional study demonstrated that the Arg406Trp variant resulted in impaired desmin assembly and destabilized filamentous networks *in vitro*, as shown by immunofluorescence microscopy. Moreover, knock-in mice carrying the Arg406Trp variant developed both myopathy and cardiomyopathy, which were associated with severe intercalated disc derangement. These findings led to the conclusion that DES Arg406Trp is causative for desmin-related myopathy (Herrmann et al., 2020). Given all indications, the variant was classified as “pathogenic” based on the American College of Medical Genetics (ACMG) guidelines (Table 1). The heterozygous state of the c.1216C>T variant was confirmed through Sanger sequencing (Figure 2A), and a diagnosis of desmin-related myopathy was concluded, consistent with the patient’s physical complaints. The two detected *TTN* variants were both classified as variants of uncertain significance (VUS) (Table 1).

**FIGURE 3:**
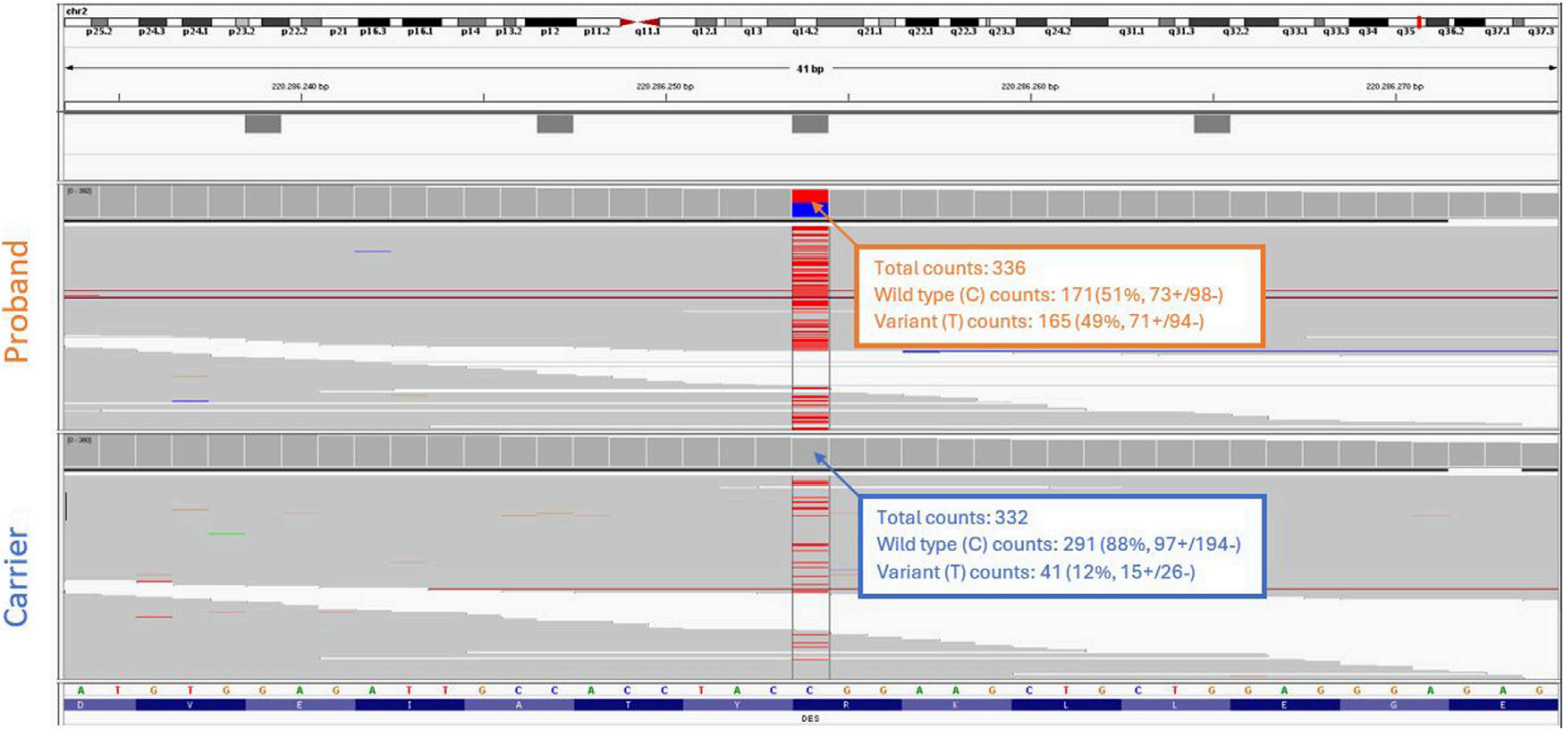
Whole exome sequencing analysis. Integrative genomics viewer (IGV) screenshot shows the NM_001927.4 (*DES*):c.1216C>T variant present in heterozygous form in the proband (top), and in mosaicism in the blood of the proband’s asymptomatic mother (bottom).

Considering the incomplete penetrance and variable expressivity of DES variants, cascade testing was initiated performing targeted Sanger sequencing on the proband’s asymptomatic parents. While the proband’s father (Figure 1, II-1) carried only the wild-type allele (Figure 2B), the mother (Figure 1, II-2) showed a slight but reproducible T signal elevation at c.1216 in the electropherogram (Figure 2C). Suspecting mosaicism, WES on maternal blood DNA (coverage: 332x) confirmed the C>T variant in 12% (41/332) of reads, establishing her mosaic status (Figure 3). To further assess mosaicism in other tissues, we collected two additional buccal-swab samples and performed WES as described above. The c.1216C>T variant was detected at mosaic levels of 4% (4/228 reads) and 15% (30/197 reads) in the two samples, respectively (Supplementary Figure S1).

Further cascade testing via targeted Sanger sequencing of the proband’s younger asymptomatic siblings showed that neither had inherited the c.1216C>T variant (Figure 1, III-2 and III-3; Supplementary Figure S2).

### 3.2 Follow-up

Following diagnosis of desmin-related myopathy, the proband was referred to a neurologist and pneumologist, as neuromuscular and respiratory involvement can be expected in patients with desmin-related myopathy. During the neurological consultation it became apparent that the patient had engaged in regular recreational strength training since the age of 16. However, following cessation of this activity at the age of 21, she began to experience progressive difficulties in daily activities. She reported a slowing of her walking pace, increased fatigue, and a decline in proximal muscle strength of the lower limbs. Additionally, she noted difficulties with articulation and swallowing. Upon neurological examination, the patient demonstrated signs of proximal and distal muscle weakness, most prominently in the lower limbs. She displayed a bilateral steppage gait, and difficulties rising from a seated position. There was noticeable weakness of the facial muscles and the neck flexors. Her speech was dysarthric, with a hyper nasal speech pattern. During a pneumological consultation, the patient reported shortness of breath with minimal exertion, such as climbing two flights of stairs. These symptoms also began following the interruption of her fitness routine at the age of 21. Pulmonary function testing revealed a restrictive pattern with associated respiratory muscle weakness as confirmed by a reduced Maximum Inspiratory Pressure (MIP) and Maximum Expiratory Pressure (MEP). Further evaluation with combined oximetry and capnography demonstrated a nocturnal alveolar hypoventilation for which home non-invasive ventilation was initiated.

The proband was administered to a multidisciplinary neuromuscular reference centre for further treatment. At the time of writing, the proband attends regular cardiologic, neurologic, psychological, pneumological, and physiotherapeutic follow-up for her symptoms, with her current status being stable based on both anamnestic and clinical examination.

Her mother, who is a mosaic carrier, underwent neurophysiological, cardiac, and pulmonary examination. All showed no apparent symptoms or clinical signs indicative of desmin-related myopathy.

## 4 Discussion

A substantial number of disease-causing variants in *DES* have been documented, with a current total of 164 variants labelled “(likely) pathogenic” in the Clinvar database and 153 variants labelled “damaging” in the Human Gene Mutation Database (Qiagen, Germany). These include variants leading to premature termination of translation (frameshift and stopgain) as well as splice variants and missense variants. The Arg406Trp variant is a well-known pathogenic variant that has both been described in symptomatic patients and functionally characterized as damaging to the DES protein using *in vitro* and *in vivo* models (Dalakas et al., 2000; Park et al., 2000; Dagvadorj et al., 2004; Herrmann et al., 2020).

An important phenomenon complicating genetic diagnostics, not only in desmin-related myopathy but in many genetic conditions, is mosaicism. Mosaicism occurs when a genetic variant is present and/or expressed in only a subset of an organism’s cells (Biesecker and Spinner, 2013), and has been described in several types of myopathy including those related to aberrations in MYH7 (Bader et al., 2022), LMNA (Wang et al., 2023), RYR1 (Estévez-Arias et al., 2024), TPM2 (Tasca et al., 2013), ACTA1 (Miyatake et al., 2014; Lehtokari et al., 2024), and collagen VI-related proteins (Armaroli et al., 2015; Donkervoort et al., 2015). A mosaic carrier may appear asymptomatic or display a milder phenotype, making the diagnosis more challenging. Detecting low-grade mosaic variants through targeted Sanger sequencing of DNA extracted from peripheral blood can be difficult, as these variants may be absent or present at such low levels in the blood cells’ genetic material that they go undetected. Additionally, low-grade mosaic variants can be missed due to the limitation of the performed test (Rohlin et al., 2009).

The diagnosis of mosaic carriers is crucial, as missing such cases has significant implications. When mosaics are overlooked, not only is there a lack of clinical follow-up for the carrier—who may still develop symptoms later in life—but the diagnosis may also be missed for siblings. This can result in incorrect recurrence risk counselling for future offspring and, more critically, the missed opportunity for prenatal testing (Rahbari et al., 2016; Biesecker and Spinner, 2013). If the variant is present in the germ cells, it can be transmitted to offspring, leading to disease in the next-generation. Failing to trace the variant back to an asymptomatic parent may lead to the erroneous conclusion of *de novo* emergence, preventing necessary actions for the family, such as further genetic testing and family planning (Rahbari et al., 2016). Therefore, accurately diagnosing mosaicism is essential for comprehensive care and genetic counselling.

Additional genetic analyses of buccal-swab DNA also revealed mosaicism for the c.1216C>T variant, indicating that this carrier harbours the variant at varying levels in tissues beyond peripheral blood. However, a limitation of this study is the lack of data on the presence of the c.1216C>T variant in cardiac tissue of the asymptomatic mother. Given the early-onset nature of desmin-related cardiomyopathy, which often manifests in the second or third decade of life (Goldfarb et al., 2004), her lack of clinical signs suggests either a lack of variant expression in cardiac tissue or reduced disease penetrance. A transthoracic ultrasound conducted after her daughter’s initial cardiological event showed no signs of cardiomyopathy. However, incomplete penetrance and variable expressivity could still explain her asymptomatic status. No heart biopsy was performed, as this would neither confirm the diagnosis nor be advisable in an asymptomatic individual (Clemen et al., 2013). Additionally, neurological examination, including electromyography, revealed no signs of myopathy. Given these factors, regular cardiological and neurological follow-up is important for the mother, as she may still develop symptoms at a later age.

Given that the variant was detected in a heterozygous state in the blood of the proband, it indicates that the c.1216C>T variant was present in the germline of the mother, leading to the inheritance. Interestingly, the mosaic variant identified here is a C>T transition in a CG sequence. In somatic *APC* mosaicism, a higher occurrence of C>T transitions in patient cases with mosaicism compared to non-mosaic cases has been described (Hes et al., 2008).

In addition to the *DES* c.1216C>T variant, we identified two missense VUS in the *TTN* gene. While *TTN* is a known causative gene for myofibrillar myopathy, the missense VUS identified in this case do not meet the criteria for a (likely) pathogenic variant based on current ACMG guidelines. Most known (likely) pathogenic *TTN* variants constitute a premature termination of translation (frameshift, stopgain) or splicing aberration with only a handful of missense variants. *TTN* missense variants are common in the general population with over 60.000 variants identified in the 1000 Genomes Project, obscuring their pathogenic potential (Jolfayi et al., 2024). However, we acknowledge that *TTN* missense variants can contribute to the phenotype in some individuals, either as a primary or secondary factor, as cases have been described (Domínguez et al., 2023). In this case, the role of the *TTN* missense VUS remains unclear. Strictly speaking they could potentially contribute to the clinical presentation in combination with the pathogenic *DES* variant, especially given the overlapping features of myofibrillar myopathy and the known variable expressivity of both genes. However, given the monogenic nature of DES related diseases, they could very well be passenger variants. Further functional studies and larger cohort analyses are needed to better understand the clinical relevance of such *TTN* VUS variants in myofibrillar myopathy (Pfeffer et al., 2014).

Another point highlighted by this case is the importance of cascade testing, even when relatives are asymptomatic, and *de novo* emergence is expected. In this case, the *DES* c.1216C>T variant had been mainly described as a *de novo* event in previous cases (Dalakas et al., 2000; Park et al., 2000; Dagvadorj et al., 2004), however, testing of the asymptomatic parents revealed the mosaic status of the pathogenic variant in the mother. This finding yielded an important indication to also test the younger, at that moment asymptomatic, siblings. One could have assumed neither of the parents carried the pathogenic c.1216C>T variant due to them being asymptomatic at advanced age, while desmin-related myopathy is known to have onset early in life, and therefore not test them, leading to a potential missed inheritance to other siblings and/or future offspring. Nevertheless, the likelihood of recurrence in siblings after a seemingly sporadic mutation varies by gene and relies on empirical data, which is often unavailable. In these cases, a risk estimation of 1%–2% is appropriate, and cascade testing in case of a presumed *de novo* variant to exclude the possibility of mosaic carriership is strongly recommended (Rahbari et al., 2016).

A prominent issue that needs to be considered hereby is the limitation of Sanger sequencing to detect mosaicism. As it depends on the intensity of the fluorescent signal in the electropherogram, low grade mosaicism could easily be missed (Rohlin et al., 2009). This shortcoming can be overcome by applying NGS techniques, such as WES, as these are able to detect DNA variants at low allele frequencies (Rohlin et al., 2009; Erickson, 2014; Qin et al., 2016). With the continuously lowering cost of NGS, this has become a valid alternative to Sanger sequencing. However, the current tendency to sequence at lower coverage poses challenges to detect mosaic variants. In clinical diagnostics, a coverage of 150x is routinely utilized when performing WES. As WES library preparation usually contains a PCR-based amplification step to enrich the captured exonic sequences, a significant number of duplicate reads are generated during sequencing. Depending on the grade of mosaicism and the depth of sequencing, the relevant variant might therefore be missed. Also, with the ongoing transition from WES to whole genome sequencing (WGS), this will become even more pronounced as WGS coverages are routinely 30–42x. Although WGS omits the need for PCR-amplification during library preparation, sequencing at sufficiently high coverage will still be essential to detect low-grade mosaicism. Furthermore, to confirm mosaicism, it is advisable to perform the analysis on several tissue types such as oral mucosa or skin biopsies. In the case presented here, this was not pursued as the carrier showed no clinical indications of disease. Finally, one other important caveat that must be taken into consideration is that the mosaic variant must be present in the blood when performing routine germline analyses, otherwise only tissue-specific analysis will be able to detect a mosaic carriership.

In conclusion, to our knowledge, this report offers the first documented confirmed case of a pathogenic *DES* variant harboured in mosaicism in the blood of an asymptomatic individual. Additionally, this report describes the first documented case of desmin-related myopathy caused by inheritance of this mosaic pathogenic *DES* variant. Furthermore, we stress both the importance of cascade testing of asymptomatic probands, even when *de novo* occurrence is suspected, and the limitations of targeted Sanger sequencing in detecting low-grade mosaicism.

## Data Availability

The original contributions presented in the study are included in the article/Supplementary Material, further inquiries can be directed to the corresponding authors.

## Supplementary material

SUPPLEMENTARY FIGURE S1 Whole exome sequencing analysis of additional tissues of mosaic carrier. Integrative genomics viewer (IGV) screenshot shows the mosaic status of the NM_001927.4(DES):c.1216C>T variant in two mouth mucosa samples of the asymptomatic carrier.

SUPPLEMENTARY FIGURE S2 Electropherograms of the proband’s siblings. Targeted Sanger sequencing of exon 6 of the desmin gene (*DES)* with position c.1216 marked. The NM_001927.4(*DES*):c.1216C>T variant is absent in the proband’s male siblings **(A, B)**.

## Abbreviations

ACMG, Americal College of Medical Genetics; MEP, Maximum Expiratory Pressure; MIP, Maximum Inspiratory Pressure; NGS, Next-generation sequencing; VUS, Variant of unknown significance; WES, Whole exome sequencing; WGS, Whole genome sequencing.
