# Supplementary figures and images for: Case Report: A first case of desmin-related myofibrillar myopathy due to inheritance from a confirmed mosaic asymptomatic carrier
Source: Front Genet. 2025 Jun 18;16:1597851. doi: 10.3389/fgene.2025.1597851 (PMC12213651; doi:10.3389/fgene.2025.1597851)

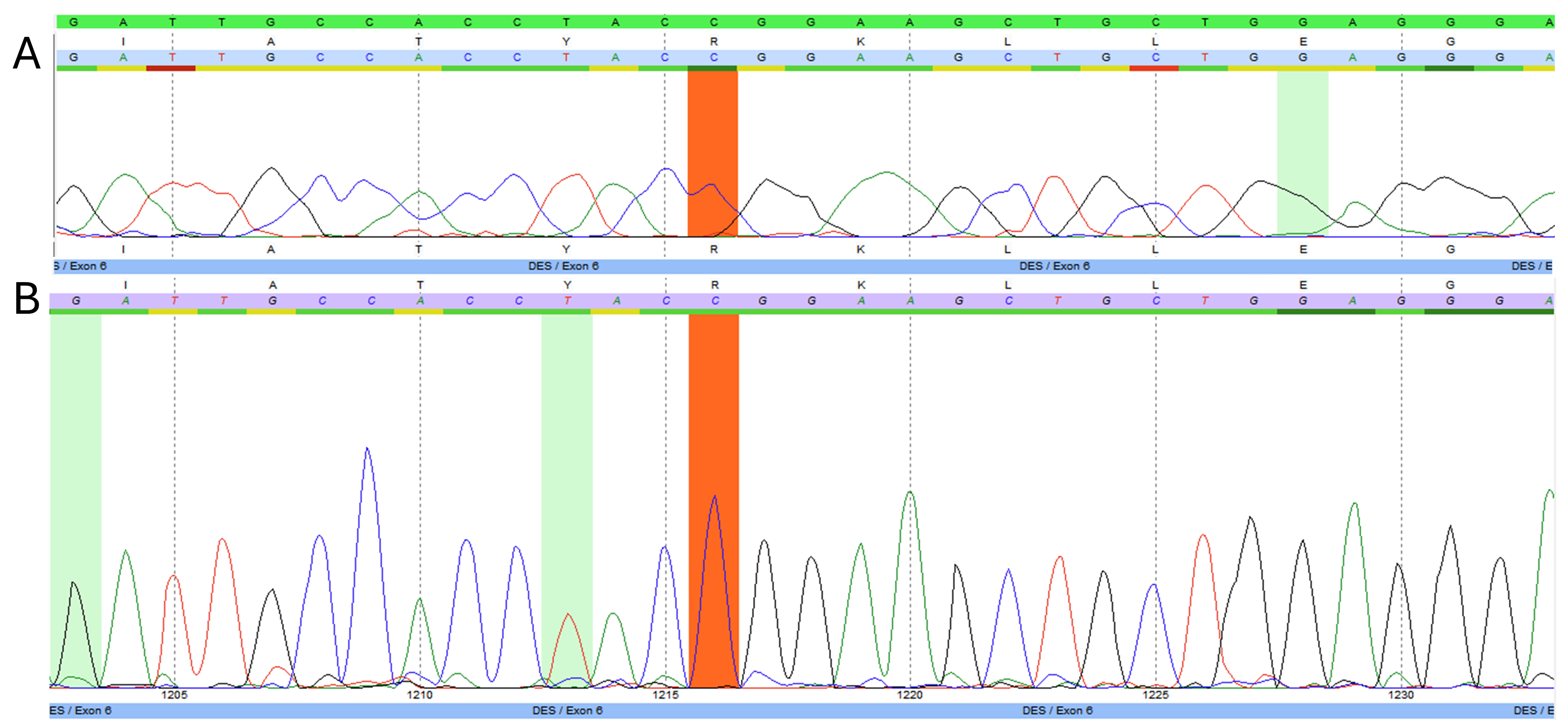

Supplement: Supplementary file 1 [file Image2.tif]

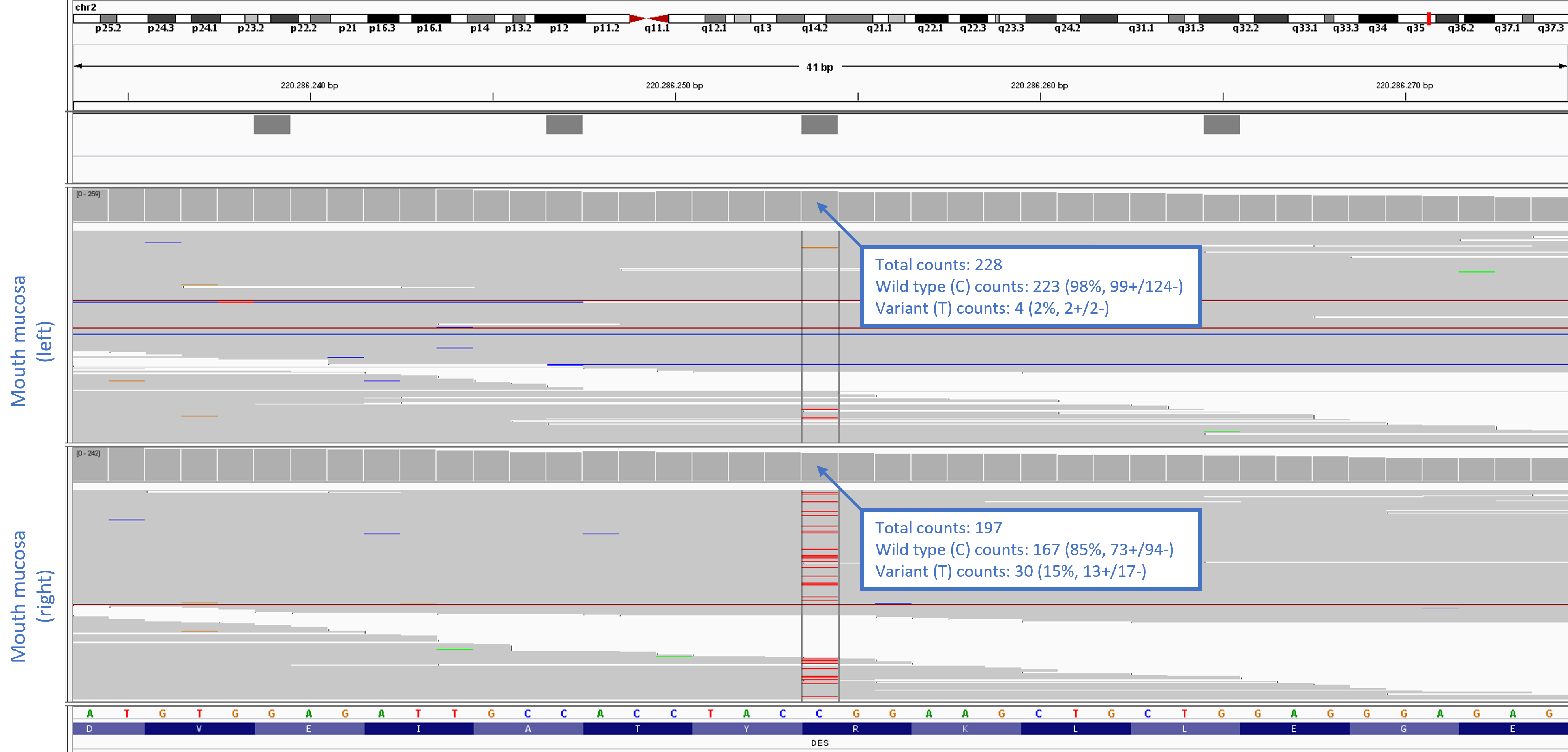

Supplement: Supplementary file 2 [file Image1.tif]
